# Association of 3‐Day Postoperative Blood Glucose Variability With All‐Cause Mortality in ICU Patients After Surgical Repair of Acute Type A Aortic Dissection: A Retrospective Cohort Study Using the MIMIC‐IV Database

**DOI:** 10.1002/clc.70381

**Published:** 2026-06-15

**Authors:** Tianyi Gu, Yingying Zhang, Gang Li, Zhonghao Pang, Jingjing Huang, Zhou Zhou, Shengjie Chen

**Affiliations:** ^1^ Department of Cardiothoracic Surgery Affiliated Hospital of Jiangsu University Zhenjiang Jiangsu China; ^2^ Department of Critical Care Medicine The First Affiliated Hospital of Anhui Medical University Hefei Anhui China; ^3^ Department of Neurosurgery Affiliated People's Hospital of Jiangsu University Zhenjiang Jiangsu China

**Keywords:** blood glucose, coefficient of variation, glucose variability, restricted cubic spline, Stanford type A aortic dissection

## Abstract

**Background:**

Stanford type A aortic dissection (TAAD) remains associated with substantial mortality despite prompt surgical repair. Glucose variability has been associated with mortality in intensive care unit (ICU) patients. However, its clinical significance after open TAAD repair remains unclear.

**Methods:**

We conducted a retrospective cohort study using the MIMIC‐IV v3.1 database (2008–2022). Glucose variability was assessed using the coefficient of variation (%CV) of blood glucose measurements within the first 3 days of postoperative ICU admission. Associations between 3‐day postoperative %CV and outcomes were evaluated using Kaplan–Meier (K–M) analysis, Cox proportional hazards (PH) models, and restricted cubic spline (RCS) analyses.

**Results:**

A total of 201 adult survivors of TAAD open repair surgery who were subsequently admitted to the ICU were included in the study. In the fully adjusted model, each 1‐standard deviation (SD) increase in the 3‐day postoperative %CV was independently associated with higher ICU mortality (adjusted hazard ratio [aHR] = 1.820, 95% confidence interval [CI]: 1.239–2.675, *p* = 0.0023) and all‐cause in‐hospital mortality (aHR = 1.627, 95% CI: 1.134–2.334, *p* = 0.0082). RCS analyses showed a positive association between the 3‐day postoperative %CV and each outcome, consistent with an approximately linear pattern. In addition, the 3‐day postoperative %CV showed stronger discriminatory ability for both outcomes than the 3‐day mean and median glucose levels.

**Conclusions:**

Higher 3‐day postoperative glucose variability was independently associated with increased all‐cause ICU mortality and in‐hospital mortality after open TAAD repair. Postoperative glucose variability may reflect postoperative physiological instability in this high‐risk population.

## Graphical abstract

Among 201 ICU patients undergoing open repair for Stanford type A aortic dissection (TAAD), higher 3‐day postoperative glucose coefficient of variation (%CV) was positively associated with ICU and in‐hospital mortality. %CV outperformed mean and median glucose and improved predictive performance when combined with the APACHE II score.

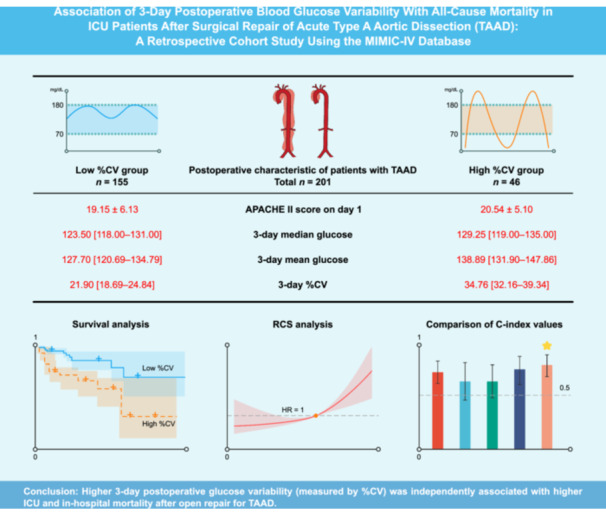

## 1 Introduction

Aortic dissection is a life‐threatening cardiovascular emergency in which an intimal tear allows blood to enter the aortic media, resulting in separation of the aortic wall layers and formation of a false lumen [1]. Once initiated, the dissection may rapidly propagate and compromise perfusion to vital organs. Dissections involving the ascending aorta (Stanford type A aortic dissection, TAAD) are particularly dangerous, with mortality approaching 50% within 48 h in the absence of prompt surgical repair [2]. Acute aortic syndrome, which includes aortic dissection, intramural hematoma, and penetrating atherosclerotic ulcer, has an incidence of approximately 3.5–6.0 per 100 000 person‐years in the general population and predominantly affects older adults [3]. Compared with type B aortic dissection (TBAD), TAAD is more common, accounting for approximately two‐thirds of acute aortic dissection cases [4, 5], and is associated with markedly higher early mortality, reaching up to 1% per hour in the absence of prompt treatment [6, 7].

Hypertension is the most important predisposing factor for aortic dissection, being present in approximately 80% of affected individuals and accounting for a substantial proportion of the population‐attributable risk [8]. Other established risk factors include advanced age, male sex, obesity, bicuspid aortic valve, connective tissue disorders such as Marfan syndrome, and smoking [9]. Because TAAD involves the ascending aorta and requires urgent definitive treatment, open surgical repair remains the standard of care for most patients [10]. In parallel with these mechanical and hemodynamic considerations, metabolic factors may also influence disease susceptibility and postoperative prognosis in complex ways. Patients with diabetes tend to develop smaller aneurysms that grow more slowly, and they experience lower aneurysm rupture‐related mortality than their non‐diabetic counterparts [11, 12]. However, diabetes does not appear to clearly alter in‐hospital or short‐term mortality following open TAAD repair, as evidenced by analyses of large readmission databases [13]. These observations suggest that chronic glycemic status may have a nuanced relationship with acute aortic disease and postoperative outcomes.

Glucose variability has consistently been associated with mortality in nondiabetic patients in the intensive care unit (ICU) [14]. The blood glucose coefficient of variation (%CV) is a commonly used measure of short‐term glycemic fluctuation. Previous studies have shown that greater postoperative glucose variability is associated with higher risks of major adverse events and mortality after coronary artery bypass grafting and in other critically ill populations [15]. Although type 2 diabetes is a recognized risk factor for many cardiovascular complications [16], the clinical significance of postoperative glucose variability in patients undergoing open repair for TAAD has not been well characterized. To address this gap, we performed a retrospective cohort study based on data collection from the Medical Information Mart for Intensive Care (MIMIC)‐IV database to investigate the associations between 3‐day postoperative blood glucose variability, measured by %CV, and ICU mortality and in‐hospital mortality in patients undergoing open surgical repair for TAAD.

## 2 Methods

### 2.1 Data Source and Study Design

This retrospective cohort study used data from version 3.1 of the Medical Information Mart for Intensive Care IV (MIMIC‐IV v3.1) database, which is maintained by the Laboratory for Computational Physiology at the Massachusetts Institute of Technology (MIT) and publicly accessible via PhysioNet (https://physionet.org/content/mimiciv/3.1/) [17, 18]. MIMIC‐IV contains detailed information on ICU admissions, including patient demographics, diagnoses coded using the International Classification of Diseases, Ninth and Tenth Revisions, Clinical Modification (ICD‐9/10‐CM), vital signs, laboratory test results, medication administration records, and clinical outcomes. This study was conducted in accordance with the Strengthening the Reporting of Observational Studies in Epidemiology (STROBE) statement [19].

### 2.2 Inclusion and Exclusion Criteria

Adult patients (≥ 18 years) diagnosed with TAAD were included if they had open cardiac surgery at the Beth Israel Deaconess Medical Center (BIDMC) between 2008 and 2022. TAAD was identified using ICD‐9 codes 441.00, 441.01, 441.02, and 441.03, as well as ICD‐10 codes I71.0, I71.01, I71.02, and I71.03. Only patients who survived the operative procedure and were admitted to the ICU after surgery were eligible for inclusion. In patients who underwent multiple eligible surgeries during their hospital stay, only the first procedure was included.

Patients were required to have at least three postoperative glucose measurements recorded within the first 3 days of postoperative ICU admission; for patients with an ICU stay of less than 3 days, all glucose measurements obtained during the ICU stay were considered. Glucose measurements could come from whole blood glucose, venous serum glucose, or fingertip capillary glucose obtained during routine clinical care. Missing values for height, weight, and laboratory variables were imputed using the median. We also excluded solid organ transplant and bone marrow transplant patients and pregnant or postpartum women.

### 2.3 Definitions

Participants with a body mass index (BMI) of 25 kg/m^2^ or higher were classified as overweight or obese. Acute kidney injury (AKI) was diagnosed according to the Kidney Disease: Improving Global Outcomes (KDIGO) guidelines [20] if any of the following criteria were met: an increase in serum creatinine of at least 0.3 mg/dL within 48 h, an increase in serum creatinine to at least 1.5 times the baseline within 7 days, or a urine output of less than 0.5 mL/kg/h for at least 6 h. Baseline creatinine was defined as the lowest creatinine value recorded within the previous 48 h or during the subsequent 7 days. If no such creatinine value was available, the first creatinine measurement obtained after hospital admission was used as the baseline. Postoperative sepsis was identified according to Sepsis‐3 consensus definition [21].

### 2.4 Measures

#### 2.4.1 Outcomes

The study endpoints were ICU mortality and all‐cause in‐hospital mortality.

#### 2.4.2 Exposure

The exposure variable was early postoperative glucose variability, defined as the coefficient of variation (%CV) of all recorded glucose measurements within the first 3 days of postoperative ICU admission. This indicator was calculated as the standard deviation (SD) of all glucose readings divided by the mean glucose level and multiplied by 100.

Glucose measurement records were extracted from multiple clinically used specimen types, including whole blood glucose, venous serum glucose, and fingertip capillary glucose. These measurements were pooled and analyzed together to reflect overall postoperative glycemic fluctuation during routine clinical care. To reduce the influence of implausible or nonphysiological values, whole blood glucose and venous serum glucose measurements greater than 1000 mg/dL were excluded, and fingertip capillary glucose measurements greater than 500 mg/dL were also excluded, according to the data‐cleaning approach described in previous literature [22]. Detailed definitions and extraction procedures for glucose‐related variables are provided in Supporting Information S1: Table 1.

For descriptive analyses and Kaplan–Meier (K–M) analyses, patients were additionally categorized into low and high 3‐day postoperative %CV groups using a data‐driven cutoff identified with the “survminer” package [23]. Because this cutoff was internally derived, 3‐day postoperative %CV was also analyzed as a continuous variable in multivariable Cox models and restricted cubic spline (RCS) analyses.

The time window length was selected for several reasons. First, the first 3 days following postoperative ICU admission represent a period of substantial physiological stress after open TAAD repair, during which glycemic fluctuations may reflect acute postoperative instability. Second, a 3‐day window provides a sufficient number of glucose measurements to allow a relatively stable assessment of variability while minimizing the influence of later ICU events unrelated to the immediate postoperative phase.

#### 2.4.3 Other Variables

For each patient, we extracted demographic and clinical variables potentially associated with postoperative prognosis, including age, sex, race, height, weight, smoking and alcohol use history, ICU and hospital length of stay (LOS), ICU and in‐hospital mortality, use of cardiopulmonary bypass (CPB) or extracorporeal membrane oxygenation (ECMO) during open surgery, the Acute Physiology and Chronic Health Evaluation II (APACHE II) [24] and Charlson Comorbidity Index (CCI) [25, 26] scores on day 1 after postoperative ICU admission, median and mean blood glucose values obtained within the first 3 postoperative days, and the first recorded heart rate, platelet count (PLT), and serum sodium level within the first 3 days of postoperative ICU admission. We also collected data on AKI, postoperative sepsis, underlying comorbidities (diabetes, hyperlipidemia, and hypertension), and the administration of insulin and glucocorticoids within 24 h after postoperative ICU admission. The CCI score was calculated from hospital discharge diagnoses, radiology notes, and past medical history (PMH). Covariates included in the adjusted models were age group (< 65 or ≥ 65 years), sex, overweight/obesity, smoking history, heart rate, PLT, serum sodium, sepsis, and CCI and APACHE II scores.

### 2.5 Statistical Analysis

Baseline demographic characteristics, laboratory parameters, and comorbidities were compared between the low and high 3‐day postoperative %CV groups using descriptive statistics. Categorical variables were analyzed using the *χ* ^2^ test or Fisher's exact test and are presented as frequencies or numbers (percentages). Continuous variables were analyzed using Student's *t*‐test or the Mann–Whitney *U* test and are presented as the mean ± standard deviation (SD) or median [Q1–Q3]. The Shapiro–Wilk test was performed to evaluate the normality of the data distribution. Absolute standardized mean differences (ASMDs), generated using the “tableone” package [27], were used to compare group differences. An ASMD of less than 0.2 was considered to indicate a small difference.

K–M curves were used to estimate and compare time‐to‐event distributions for ICU mortality and all‐cause in‐hospital mortality between the low and high 3‐day postoperative %CV groups. Survival curves were compared using the log‐rank test. To evaluate the associations between exposure and outcomes, we fitted univariable and multivariable Cox proportional hazards (PH) models and reported hazard ratios (HRs) with 95% confidence intervals (CIs). Glucose measurements from whole blood, venous serum, and fingertip capillary samples were pooled for the calculation of 3‐day postoperative mean glucose and 3‐day postoperative %CV. Median glucose values during the same time window were also extracted. The PH assumption was assessed for both the exposure variable and the covariates in the fully adjusted models. Cox PH regression analyses and concordance index (C‐index) calculations were conducted using the “survival” package [28].

RCS analyses were performed to explore the nonlinear associations between 3‐day postoperative %CV as a continuous variable and the risks of ICU mortality and all‐cause in‐hospital mortality. Because of the limited sample size, the number of knots was set to three to ensure adequate observations between knots for stable estimation [29]. RCS plots were generated using the “rcssci” [30, 31] and “ggplot2” packages [32].

Relative excess risk due to interaction (RERI) and attributable proportion due to interaction (AP) [33] were used to explore the biological interaction between 3‐day postoperative %CV and selected comorbidities. These analyses were conducted using the “interaction” package [34] and the “RcmdrMisc” package [35].

All statistical tests were two‐sided. A *p*‐value < 0.05 was considered statistically significant. All statistical analyses were performed using R software (version 4.4.0 for Windows; R Foundation for Statistical Computing, Vienna, Austria).

### 2.6 Ethical Considerations

This study was conducted in accordance with the Declaration of Helsinki. The MIMIC‐IV database was approved by the Institutional Review Boards of BIDMC (2001‐P‐001699/14) and MIT (0403000206), both of which waived informed consent. Only de‐identified data were used in this study, and patient confidentiality was maintained throughout. Author Zhou Zhou obtained permission to access the database (Record ID 11493928) and performed the data extraction.

## 3 Results

**FIGURE 1:**
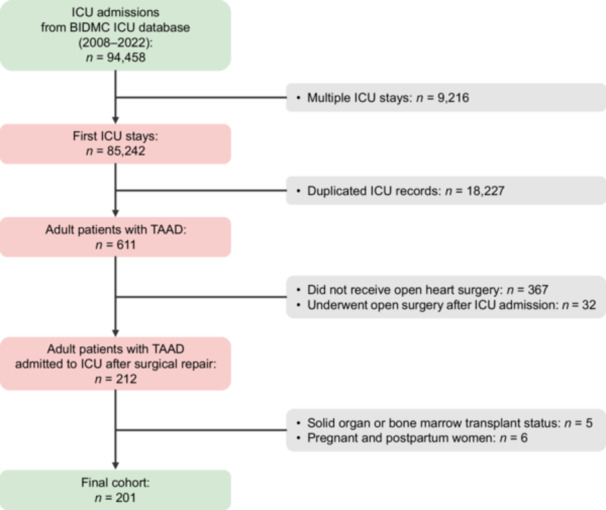
Consolidated Standards of Reporting Trials (CONSORT) diagram of cohort creation and identification of eligible patients from the MIMIC‐IV database for inclusion.

Between 2008 and 2022, 611 adult patients with acute TAAD were admitted to the ICU. After the predefined exclusion criteria were applied, 201 adult survivors who underwent open TAAD repair and were subsequently admitted to the ICU were included in the final analysis (Figure 1).

### 3.1 Patient Characteristics and Outcomes

Among the 201 patients with TAAD who underwent open surgical repair, the mean age was 64.15 ± 13.73 years, and 69.65% were male. Only 13 patients (6.47%) did not develop AKI within 7 days after surgery. The optimal cutoff value of 3‐day postoperative %CV, determined by maximizing the between‐group difference in in‐hospital survival, was 30.08849. Accordingly, patients were classified into low 3‐day postoperative %CV group (*n* = 155) and high 3‐day postoperative %CV group (*n* = 46). Detailed demographic and baseline characteristics, as well as clinical outcomes, of the patients are shown in Supporting Information S1: Table 2 and summarized in Table 1.

**TABLE 1:** Comparison of demographic and baseline characteristics and clinical outcomes by postoperative glucose variability group.

Characteristic	Total	Low %CV (%CV < 30.08849)	High %CV (%CV ≥ 30.08849)	*p*	ASMD
*n* (%)	201	155 (77.11)	46 (22.89)		
Age (yr), mean ± SD	64.15 ± 13.73	63.56 ± 13.94	66.13 ± 12.96	0.2661	0.191
Age ≥ 65 yr, *n* (%)	99 (49.25)	74 (47.74)	25 (54.35)	0.4313	0.132
Male, *n* (%)	140 (69.65)	113 (72.90)	27 (58.70)	0.0657	0.303
Race, *n* (%)				0.0135	0.620
Caucasian	109 (54.23)	91 (58.71)	18 (39.13)		
Latino	10 (4.98)	5 (3.23)	5 (10.87)		
Asian	8 (3.98)	8 (5.16)	0 (0.00)		
Black	17 (8.46)	13 (8.39)	4 (8.70)		
Other/unknown	57 (28.36)	38 (24.52)	19 (41.30)		
Overweight/obese (BMI ≥ 25 kg/m^2^), *n* (%)	148 (73.63)	116 (74.84)	32 (69.57)	0.4760	0.118
Smoking history, *n* (%)	66 (32.84)	48 (30.97)	18 (39.13)	0.3006	0.172
Alcohol consumption, *n* (%)	18 (8.96)	15 (9.68)	3 (6.52)	0.7157	0.116
CCI score, median [Q1–Q3]	4.00 [3.00–5.00]	4.00 [3.00–5.00]	4.00 [3.00–5.00]	0.6450	0.066
APACHE II score on day 1, mean ± SD	19.47 ± 5.93	19.15 ± 6.13	20.54 ± 5.10	0.1259	0.246
Use of CPB/ECMO during surgery, *n* (%)	195 (97.01)	151 (97.42)	44 (95.65)	0.9004	0.097
3‐day median glucose (mg/dL), median [Q1–Q3]	124.50 [118.00–132.50]	123.50 [118.00–131.00]	129.25 [119.00–135.00]	0.0380	0.335
3‐day mean glucose (mg/dL), median [Q1–Q3]	130.45 [121.55–137.41]	127.70 [120.69–134.79]	138.89 [131.90–147.86]	< 0.001	0.922
3‐day %CV, median [Q1–Q3]	24.02 [19.88–29.12]	21.90 [18.69–24.84]	34.76 [32.16–39.34]	< 0.001	2.754
Blood Na^+^ (mmol/L), median [Q1–Q3]	141.00 [139.00–144.00]	141.00 [139.00–143.00]	142.00 [139.25–146.00]	0.0608	0.360
Heart rate (bpm), median [Q1–Q3]	82.00 [77.00–88.00]	80.00 [76.00–87.00]	84.00 [79.25–89.50]	0.1050	0.251
PLT (K/μL), median [Q1–Q3]	111.00 [86.00–143.00]	111.00 [88.50–142.00]	114.00 [85.00–153.75]	0.6840	0.124
Sepsis, *n* (%)	138 (68.66)	108 (69.68)	30 (65.22)	0.5669	0.095
Diabetes, *n* (%)	18 (8.96)	14 (9.03)	4 (8.70)	1.0000	0.012
Hyperlipidemia, *n* (%)	82 (40.80)	64 (41.29)	18 (39.13)	0.7935	0.044
Hypertension, *n* (%)	166 (82.59)	131 (84.52)	35 (76.09)	0.1856	0.213
Use of insulin within 3 days after surgery, *n* (%)	197 (98.01)	152 (98.06)	45 (97.83)	1.0000	0.017
Use of glucocorticoids within 3 days after surgery, *n* (%)	13 (6.47)	8 (5.16)	5 (10.87)	0.2979	0.211
AKI occurrence, *n* (%)				0.0441	0.360
Dialysis‐dependent	6 (2.99)	2 (1.29)	4 (8.70)		
Occurrence within 7 days after surgery	182 (90.55)	142 (91.61)	40 (86.96)		
No AKI occurred within 7 days after surgery	13 (6.47)	11 (7.10)	2 (4.35)		
RRT initiation, *n* (%)				0.0201	0.401
Dialysis‐dependent	6 (2.99)	2 (1.29)	4 (8.70)		
Not initiated	165 (82.09)	132 (85.16)	33 (71.74)		
Initiated after postoperative ICU admission	30 (14.93)	21 (13.55)	9 (19.57)		
ICU LOS (d), median [Q1–Q3]	4.95 [2.48–10.81]	5.22 [2.87–10.17]	3.81 [2.21–13.39]	0.4430	0.022
Hospital LOS (d), median [Q1–Q3]	10.27 [6.84–16.77]	10.30 [7.15–16.45]	8.40 [5.16–17.93]	0.2140	0.056
ICU mortality, *n* (%)	23 (11.44)	10 (6.45)	13 (28.26)	< 0.001	0.601
In‐hospital mortality, *n* (%)	25 (12.44)	12 (7.74)	13 (28.26)	< 0.001	0.554

Abbreviations: AKI, acute kidney injury; APACHE II, Acute Physiology and Chronic Health Evaluation II; ASMD, absolute standardized mean difference; bpm, beats per minute; CCI, Charlson Comorbidity Index; CV, coefficient of variation; ICU, intensive care unit; LOS, length of stay; PLT, platelet count; SD, standard deviation.

Compared with the low 3‐day postoperative %CV group, patients in the high group had significantly higher mean glucose levels and greater glucose variability (both *p* < 0.05; both ASMDs ≥ 0.2). Although APACHE II score on day 1 and heart rate were not statistically significant between groups, both were higher in the high %CV group and showed meaningful between‐group imbalance based on ASMD values (both ASMDs ≥ 0.2). No significant differences observed for age, CCI score, overweight/obesity, smoking history, alcohol consumption, diabetes, hyperlipidemia, sepsis, CPB or ECMO use during surgery, PLT, or postoperative insulin use (all *p* > 0.05; all ASMDs < 0.2).

RRT was initiated more frequently in the high 3‐day postoperative %CV group (*p* = 0.0201; ASMD = 0.401). There was no significant difference in ICU or hospital length of stay (LOS) (both *p* > 0.05; both ASMDs < 0.2). However, ICU mortality and in‐hospital mortality were significantly higher in the high %CV group (both *p* < 0.001; both ASMDs ≥ 0.2).

More than 90% of patients underwent CPB or ECMO during surgery and received insulin within the first 3 days after postoperative ICU admission. In addition, fewer than 10% of patients received glucocorticoids during the first 3 days after postoperative ICU admission or had a documented history of alcohol consumption. Owing to their limited variability, these variables were not included as covariates in the fully adjusted model.

### 3.2 Survival Analysis

**FIGURE 2:**
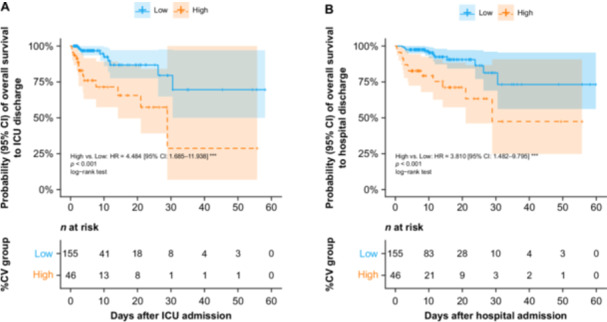
Kaplan–Meier (K–M) curves comparing the high and low 3‐day postoperative coefficient of variation (%CV) groups for (A) ICU mortality and (B) in‐hospital mortality. The shaded regions represent the 95% confidence intervals (CIs).

ICU mortality and in‐hospital mortality were evaluated in patients with TAAD who underwent open surgical repair. Comparisons of ICU mortality and in‐hospital mortality between the high and low 3‐day postoperative %CV groups are shown in Figure 2. K–M analysis showed significantly higher cumulative mortality in the high %CV group than in the low %CV group for both ICU mortality and in‐hospital mortality (both log‐rank *p* < 0.05).

Cox regression was further used to examine the association between %CV and the primary and secondary outcomes. In the fully adjusted models for ICU mortality (Supporting Information S1: Table 3) and in‐hospital mortality (Supporting Information S1: Table 4), all covariates satisfied the PH assumption (all *p* > 0.05). When %CV was analyzed as a continuous variable, higher %CV was significantly associated with an increased risk of ICU mortality. This association was significant in the crude model (crude HR [cHR] = 1.706, 95% CI: 1.192–2.441, *p* = 0.0035), after adjustment for age, sex, and BMI category (Model 1: adjusted HR [aHR] = 1.868, 95% CI: 1.254–2.783, *p* = 0.0021), and after further adjustment for smoking history, clinical covariates, and illness severity (Model 2: aHR = 1.820, 95% CI: 1.239–2.675, *p* = 0.0023). Similarly, higher %CV was significantly associated with an increased risk of in‐hospital mortality in the crude model (cHR = 1.582, 95% CI: 1.133–2.208, *p* = 0.0070), Model 1 (aHR = 1.693, 95% CI: 1.173–2.443, *p* = 0.0049), and Model 2 (aHR = 1.627, 95% CI: 1.134–2.334, *p* = 0.0082). When %CV was analyzed as a categorical variable in the crude model, patients in the high %CV group had significantly higher risks of both ICU mortality and in‐hospital mortality than those in the low %CV group (both *p* < 0.001). These results are consistent with the differences observed in the K–M curves. Furthermore, these associations remained statistically significant in Model 1 and Model 2 (all *p* < 0.01) (Table 2).

**TABLE 2:** Associations of postoperative glucose variability with ICU mortality and hospital mortality.

Group	Crude Model		Model 1		Model 2	
	cHR [95% CI]	*p*	aHR [95% CI]	*p*	aHR [95% CI]	*p*
**ICU mortality**						
Continuous variable (per 1 SD)	1.706 [1.192–2.441]	0.0035	1.868 [1.254–2.783]	0.0021	1.820 [1.239–2.675]	0.0023
Categorical variable						
Low %CV	Reference		Reference		Reference	
High %CV	4.591 [1.999–10.546]	< 0.001	5.016 [2.107–11.939]	< 0.001	7.400 [2.798–19.573]	< 0.001
**In‐hospital mortality**						
Continuous variable (per 1 SD)	1.582 [1.133–2.208]	0.0070	1.693 [1.173–2.443]	0.0049	1.627 [1.134–2.334]	0.0082
Categorical variable						
Low %CV	Reference		Reference		Reference	
High %CV	3.826 [1.743–8.396]	< 0.001	3.970 [1.775–8.876]	< 0.001	5.588 [2.305–13.545]	< 0.001

*Note:* Model 1 is adjusted for age group, sex, and BMI category. Model 2 is further adjusted for smoking history, heart rate, platelet count (PLT), blood sodium, sepsis, and Charlson Comorbidity Index (CCI) and Acute Physiology and Chronic Health Evaluation II (APACHE II) scores.

Abbreviations: aHR, adjusted hazard ratio; cHR, crude hazard ratio; CI, confidence interval; CV, coefficient of variation; SD, standard deviation.

### 3.3 Exploration of Nonlinear Associations Between 3‐Day Postoperative %CV and Outcomes

**FIGURE 3:**
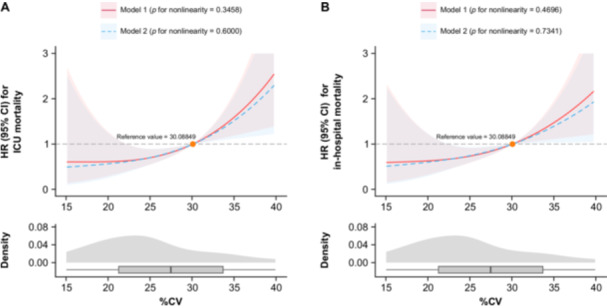
Fitted adjusted Cox regression restricted cubic spline (RCS) models and distribution of the 3‐day postoperative coefficient of variation (%CV). The RCS splines demonstrate the associations between %CV and the risk of all‐cause (A) ICU mortality and (B) in‐hospital mortality. The number of knots was three. The reference value is 30.08849. The shaded area represents the 95% CI for the HR. Model 1 is adjusted for age group, sex, and BMI category. Model 2 is further adjusted for smoking history, heart rate, platelet count (PLT), blood sodium, sepsis, and Charlson Comorbidity Index (CCI) and Acute Physiology and Chronic Health Evaluation II (APACHE II) scores. The shaded regions represent the 95% confidence intervals (CIs).

RCS with three knots fitted to Model 1 and Model 2 are presented in Figure 3. As the 3‐day postoperative glucose %CV increased, the HRs for both ICU mortality (Figure 3A) and in‐hospital mortality (Figure 3B) increased. Using the cutoff value as the reference, higher %CV values were associated with progressively greater risks of death. The tests for nonlinearity were not statistically significant for either endpoint (all *p* for nonlinearity > 0.05), suggesting that the associations between 3‐day postoperative %CV and both ICU mortality and in‐hospital mortality were approximately linear.

### 3.4 Predictive Performance of 3‐Day Postoperative %CV

**FIGURE 4:**
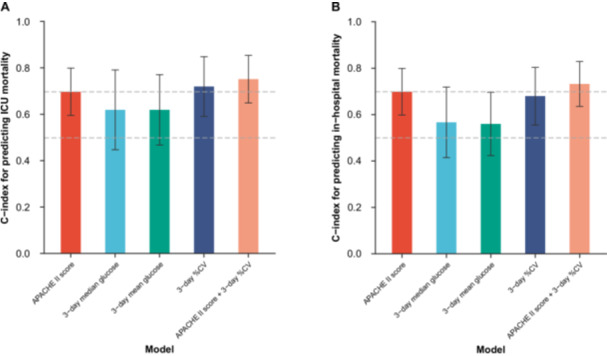
Performance of models established in the MIMIC‐IV cohort. C‐index values indicate the discriminatory ability of models for predicting (A) ICU mortality and (B) in‐hospital mortality in ICU patients with Stanford type A aortic dissection (TAAD) who underwent open surgical repair. Error bars indicate the 95% confidence intervals (CIs). CV, coefficient of variation.

Further exploratory comparisons of discriminatory performance revealed clear differences among the models (Figure 4). For both ICU mortality (Figure 4A) and in‐hospital mortality (Figure 4B), the C‐index of 3‐day postoperative %CV exceeded 0.60, indicating acceptable discriminative ability. Furthermore, the 3‐day postoperative %CV demonstrated superior discrimination compared to the APACHE II score in predicting ICU mortality. In addition, the 3‐day postoperative %CV showed better discrimination than the 3‐day mean or median glucose levels for predicting both outcomes. Moreover, the combination of the 3‐day postoperative %CV and the day‐1 APACHE II score demonstrated better predictive performance than either %CV or APACHE II score alone.

### 3.5 Biological Interaction Analysis

Potential biological interactions between 3‐day postoperative %CV and hypertension, as well as between 3‐day postoperative %CV and hyperlipidemia, were evaluated (Table 3). Neither the RERI nor the AP indicated a significant biological interaction.

**TABLE 3:** Hazard ratios (HRs) for all‐cause ICU mortality with contributions from exposure variable (3‐day postoperative coefficient of variation [%CV]) and comorbidities.

Subgroup	No. of deaths/total no. of patients (%)	HR (per 1 SD) [95% CI]	RERI [95% CI]	AP [95% CI]
Hyperlipidemia			−0.488 [−1.769–0.794]	−0.222 [−0.885–0.442]
No	14/119 (11.76%)	1.948 [1.228–3.088]		
Yes	11/82 (13.41%)	1.264 [0.777–2.057]		
Hypertension			−0.282 [−1.180–0.616]	−0.220 [−0.975–0.535]
No	6/35 (17.14%)	1.691 [0.976–2.931]		
Yes	19/166 (11.45%)	1.469 [0.949–2.276]		

Abbreviations: AP, attributable proportion due to interaction; RERI, relative excess risk due to interaction.

## 4 Discussion

In this retrospective cohort study using the MIMIC‐IV database, higher 3‐day postoperative %CV was associated with greater risks of ICU and in‐hospital mortality in patients undergoing open surgical repair for TAAD. Rather than focusing solely on absolute glucose levels or pre‐existing diabetes, our findings suggest that short‐term postoperative glucose fluctuation may be associated with early mortality after TAAD surgery and may reflect postoperative physiological instability in this setting.

Several epidemiological studies [5, 36, 37] have reported a rising incidence of acute aortic dissection in Western populations. Interestingly, diabetes mellitus has been associated with a lower risk of both aortic dissection and aneurysm formation [38]. One prior study suggested that chronic hyperglycemia may exert protective effects against aortic dissection [39]. Mechanistically, chronic hyperglycemia has been proposed to stabilize the aortic wall by downregulating matrix metalloproteinase (MMP) activity, upregulating plasminogen activator inhibitor‐1 (PAI‐1), reducing adventitial neovascularization, and enhancing transforming growth factor (TGF)‐β signaling, thereby preserving extracellular matrix integrity and limiting medial degeneration [38, 40, 41]. In addition, the chronic low‐grade inflammatory milieu associated with diabetes may promote adaptive remodeling responses that render the aortic wall less susceptible to acute tearing [42], while insulin and related growth factors may influence smooth muscle cell survival and matrix synthesis [43].

However, not all studies have found a survival advantage associated with diabetes after aortic dissection repair. For example, a Swedish nationwide population‐based matched cohort study found no significant difference in adjusted mortality up to 2 years after hospitalization for aortic dissection between individuals with type 2 diabetes and matched controls without diabetes [44]. Importantly, previous studies have largely focused on chronic glycemic status rather than dynamic perioperative glucose fluctuation. Our study extends this literature by suggesting that, in the postoperative ICU setting, glucose variability remained associated with mortality after accounting for diabetic status, although residual confounding remains possible.

The observed association between a higher 3‐day postoperative %CV and mortality is clinically plausible. The immediate postoperative period after open TAAD repair is characterized by substantial physiological stress, hemodynamic instability, inflammatory activation, and frequent organ dysfunction, all of which may contribute to greater glucose variability. In the present study, patients in the high %CV group showed higher APACHE II scores than those in the low %CV group. Although this difference was not statistically significant, the ASMD exceeded 0.2, indicating a non‐negligible baseline imbalance in illness severity. Furthermore, RCS analyses showed positive associations between the 3‐day postoperative %CV and outcomes, consistent with an approximately linear pattern.

Our findings are broadly consistent with previous studies conducted in cardiovascular and critical care settings. Increased glucose variability within the first 24 h of coronary artery bypass grafting has been associated with major adverse postoperative events [45]. Time‐weighted average glucose has likewise been linked to heightened inflammation and poorer postoperative outcomes in patients with acute aortic dissection undergoing open repair [46, 47]. Taken together, these studies suggest that dynamic glycemic indices may capture aspects of perioperative stress and metabolic dysregulation that are not fully reflected by static glucose measures.

Moreover, in the present cohort, 3‐day postoperative %CV showed a higher C‐index than the corresponding 3‐day mean and median glucose levels for predicting both outcomes, suggesting that glucose variability may reflect outcome‐related information beyond that captured by static glucose metrics alone. In addition, the combination of %CV with the APACHE II model resulted in a modest improvement in discrimination. Collectively, these findings suggest that glucose variability may capture aspects of postoperative physiological instability not fully reflected by conventional severity of illness scores.

From a clinical perspective, our findings indicate that postoperative glucose variability was associated with ICU mortality and in‐hospital mortality in patients with TAAD undergoing open surgical repair. Whether patients with marked glucose fluctuations would benefit from modified monitoring or glucose management strategies remains uncertain and requires prospective studies. However, the present study does not demonstrate that reducing glucose variability itself will improve survival. Therefore, strategies such as stricter glycemic targets, continuous glucose monitoring, or individualized insulin protocols [48] should be regarded as hypotheses for future prospective or interventional studies rather than immediate practice recommendations based on existing observational evidence.

The biological interaction analysis did not show a significant interaction between 3‐day postoperative %CV and hypertension or hyperlipidemia. These findings indicate that, within this cohort, these comorbidities did not materially alter the association between postoperative glucose variability and mortality. However, given the limited sample size, these interaction analyses should be interpreted with caution.

This study has several limitations. First, the sample size and number of events were relatively modest, which may have limited statistical power and the precision of the multivariable estimates. Second, the single‐center nature of the MIMIC‐IV critical care database may limit the generalizability of our findings to other institutions and perioperative settings. Third, despite multivariable adjustment, residual confounding is likely. Important perioperative variables, such as operative complexity, CPB/ECMO duration, intraoperative hypotension, transfusion burden, vasopressor requirements, and detailed postoperative infectious complications, were not comprehensively available in the database. Fourth, the frequency of glucose measurements may have been influenced by patient acuity, potentially introducing surveillance bias. In addition, the primary clinical implication of our findings is in postoperative risk stratification rather than in guiding therapeutic decision‐making. Future multicenter studies with larger sample sizes and more granular perioperative data are needed to validate these findings and clarify whether interventions targeting postoperative glucose variability can improve outcomes after TAAD surgery.

## 5 Conclusions

In conclusion, higher 3‐day postoperative %CV was associated with increased ICU mortality and in‐hospital mortality in patients undergoing open surgical repair for TAAD. These findings suggest that greater short‐term postoperative glucose variability is associated with higher short‐term mortality in this high‐risk population. In exploratory discrimination analyses, 3‐day postoperative %CV showed a higher C‐index than mean glucose alone in this cohort. However, whether this association can be translated into reproducible clinical risk estimation or actionable management strategies requires external validation.

## Author Contributions

Tianyi Gu contributed to conceptualization, data curation, formal analysis, and drafting of the original manuscript. Yingying Zhang contributed to methodology, data curation, and formal analysis. Gang Li contributed to methodology and data curation. Zhonghao Pang and Jingjing Huang contributed to formal analysis and drafting of the original manuscript. Zhou Zhou contributed to conceptualization, supervision, and manuscript review and editing. Shengjie Chen contributed to supervision and manuscript review and editing. All authors critically reviewed the manuscript and approved the final version for submission.

## Funding

The authors have nothing to report.

## Ethics Statement

Investigations were conducted in accordance with the Declaration of Helsinki. The collection of patient information and the creation of the research resource for publication were reviewed and approved by the Institutional Review Board of BIDMC (2001‐P‐001699/14) and MIT (0403000206).

## Consent

Institutional Review Board of BIDMC (2001‐P‐001699/14) and MIT (0403000206) waived informed consent.

## Conflicts of Interest

The authors declare no conflicts of interest.

## Supporting information


**Table S1:** Glucose measurement records of patients.
**Table S2:** Patient demographics, baseline characteristics, and clinical outcomes.
**Table S3:** Assessment of the proportional hazards (PH) assumption in a Cox regression model estimating the risk of intensive care unit (ICU) mortality.
**Table S4:** Assessment of the proportional hazards (PH) assumption in a Cox regression model estimating the risk of in‐hospital mortality.

## Data Availability

Research data are not shared. The data supporting the findings of this study are available in the manuscript and supplementary files.
